# *Helicobacter pylori*-Derived Outer Membrane Vesicles Promote Hepatocellular Carcinoma Cell Proliferation with PI3K/AKT/mTOR Activation and Cyclin D1 Upregulation

**DOI:** 10.3390/ijms27219573

**Published:** 2026-10-26

**Authors:** Jun-Peng Zhou, Xiao-Na Song, Jiao Li, Hui Huang, Han Chen, Hong Tang, Hong Li

**Affiliations:** 1 Center of Infectious Diseases, West China Hospital, Sichuan University, Chengdu 610041, China; zhoujunpeng1996@163.com (J.-P.Z.); 2024224060047@stu.scu.edu.cn (X.-N.S.);; 2 Laboratory of Infectious and Liver Diseases, Institution of Infectious Diseases, West China Hospital, Sichuan University, Chengdu 610041, China; 3 Division of Infectious Diseases, State Key Laboratory of Biotherapy and Center of Infectious Diseases, West China Hospital, Sichuan University, Chengdu 610041, China

**Keywords:** *Helicobacter pylori*, outer membrane vesicles, hepatocellular carcinoma, PI3K/AKT/mTOR, Cyclin D1, cell cycle

## Abstract

Accumulating evidence suggests an association between *Helicobacter pylori* infection and liver diseases; however, it remains unclear whether *H. pylori* or its extracellular products directly influence hepatocellular carcinoma (HCC) biology. Here, we examined the effects of viable *H. pylori*, sterile *H. pylori*-conditioned supernatant (*Hp*-CS), and isolated *H. pylori*-derived outer membrane vesicles (*Hp*-OMVs) on HCC cells. *H. pylori* adhered to and invaded Huh7 and Hepa1-6 cells, increased Cyclin D1 expression, and enhanced the growth of tumors derived from bacteria-exposed Hepa1-6 cells in immunocompetent C57BL/6J mice. *Hp*-CS increased HCC cell metabolic activity, colony formation, migration, and invasion. *Hp*-OMVs were characterized by transmission electron microscopy and nanoparticle tracking analysis. Exposure to *Hp*-OMVs increased HCC cell metabolic activity, colony formation, cell-cycle progression, and tumor growth in a syngeneic mouse model. *Hp*-OMVs also increased PI3K, AKT, and mTOR phosphorylation and elevated Cyclin D1 expression. The PI3K inhibitor LY294002 attenuated these molecular changes and reduced *Hp*-OMV-induced cell-cycle progression and colony formation. These findings show that *H. pylori* and its extracellular products promote proliferation-associated phenotypes in experimental HCC models. *Hp*-OMVs are biologically active extracellular components of *H. pylori*, and pharmacological inhibition supports the involvement of PI3K/AKT/mTOR signaling and Cyclin D1 upregulation in their growth-promoting effects.

## 1. Introduction

Liver cancer is the third leading cause of cancer-related mortality worldwide, and hepatocellular carcinoma (HCC) is the most common type of primary liver cancer [1,2]. Chronic hepatitis B or C virus infection, alcohol-related liver disease, metabolic dysfunction-associated steatotic liver disease, and aflatoxin exposure are major etiological factors for HCC [2,3]. Beyond these established etiologies, growing evidence suggests that microbial and microbe-derived factors may also influence HCC development and progression [4].

*Helicobacter pylori* is a Gram-negative bacterium that colonizes the gastric mucosa and is a well-established cause of chronic gastritis, peptic ulcer disease, gastric adenocarcinoma, and gastric mucosa-associated lymphoid tissue lymphoma [5,6,7]. Epidemiological studies and meta-analyses have reported associations between *H. pylori* infection or *Helicobacter* species detection and HCC [8,9]. Molecular studies have detected *Helicobacter* 16S rDNA or *H. pylori*-related gene fragments in liver tissues from patients with cirrhosis and HCC [10,11,12,13], whereas only a limited number of studies have reported the isolation of culturable *Helicobacter* from diseased liver tissue [14,15,16]. Thus, whether viable *H. pylori* can reach and persistently colonize the human liver remains a matter of debate, raising the possibility that bacterial products originating from the gastric site of infection may contribute to the reported hepatic effects [17,18,19].

Outer membrane vesicles (OMVs) provide a potential mechanism for such bacteria–host communication. OMVs are nanosized membrane vesicles released by Gram-negative bacteria and can protect and transport bacterial components beyond the site of bacterial colonization [20,21,22,23,24,25]. *H. pylori*-derived OMVs (*Hp*-OMVs) can interact with and be internalized by host cells, providing a potential vehicle for the delivery of bacterial signals beyond the gastric niche [23,26,27,28]. However, whether *Hp*-OMVs directly influence the biological behavior of HCC cells remains unclear.

The PI3K/AKT/mTOR pathway is frequently activated in HCC and regulates multiple processes involved in tumor growth, including cell survival, metabolism, protein synthesis, and cell-cycle progression [29,30]. Cyclin D1, a cell-cycle regulator influenced by this pathway [31], promotes the G1/S-phase transition [32,33,34], and its overexpression has been reported in HCC [35]. In gastric epithelial models, *H. pylori* activates PI3K/AKT/mTOR signaling and promotes cell survival and protein translation [36,37]; however, whether *H. pylori* can similarly activate this pathway and increase Cyclin D1 expression in HCC cells remains unclear. In this study, we used viable *H. pylori*, sterile *Hp*-CS, and isolated *Hp*-OMVs to assess their effects on HCC cell proliferation, cell-cycle progression, and tumor growth in immunocompetent syngeneic mouse models, focusing on PI3K/AKT/mTOR signaling and Cyclin D1 expression.

## 2. Results

### 2.1. Viable H. pylori Interacts with HCC Cells, and Transient Exposure Enhances Subsequent Tumor Growth In Vivo

**Figure 1:**
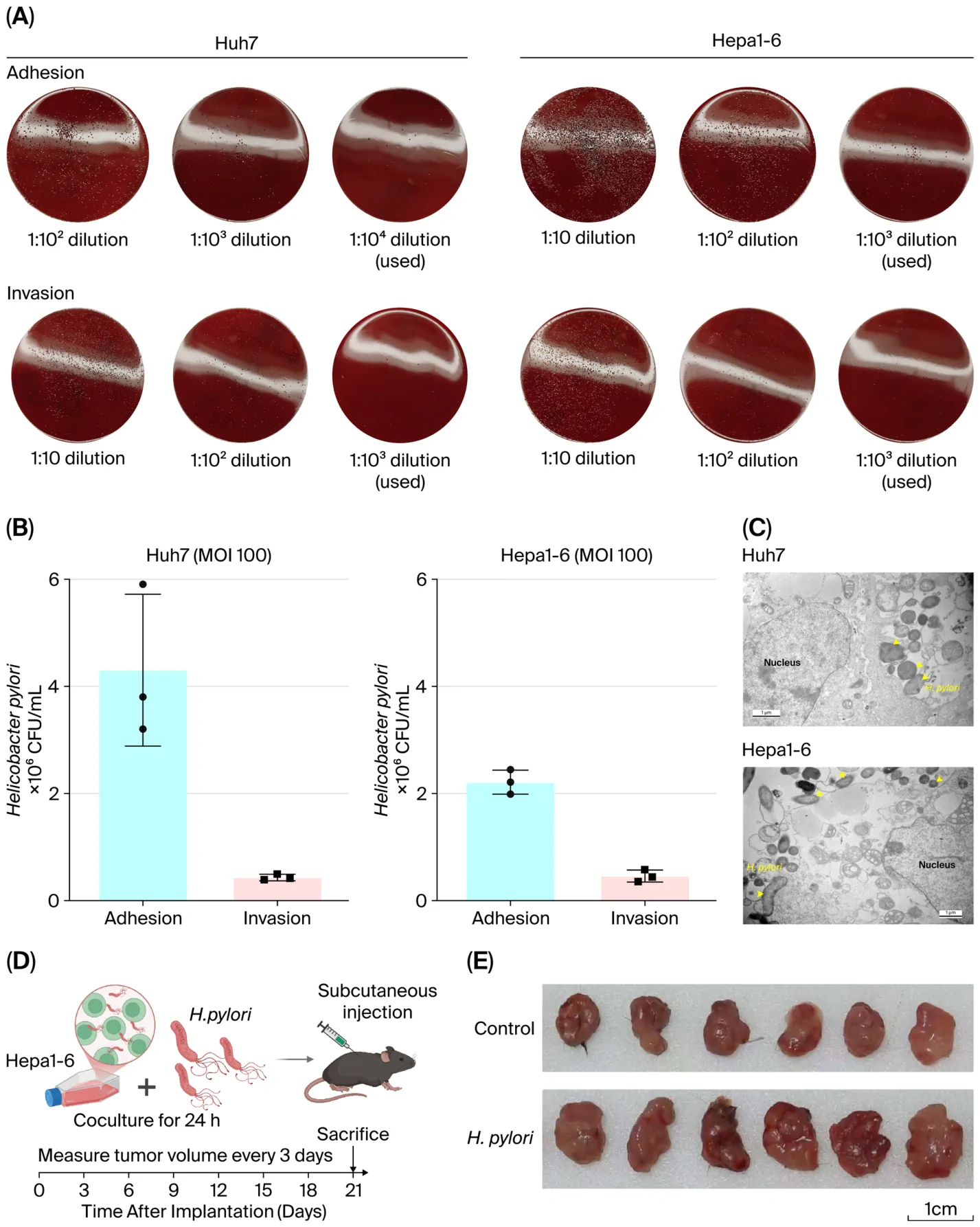

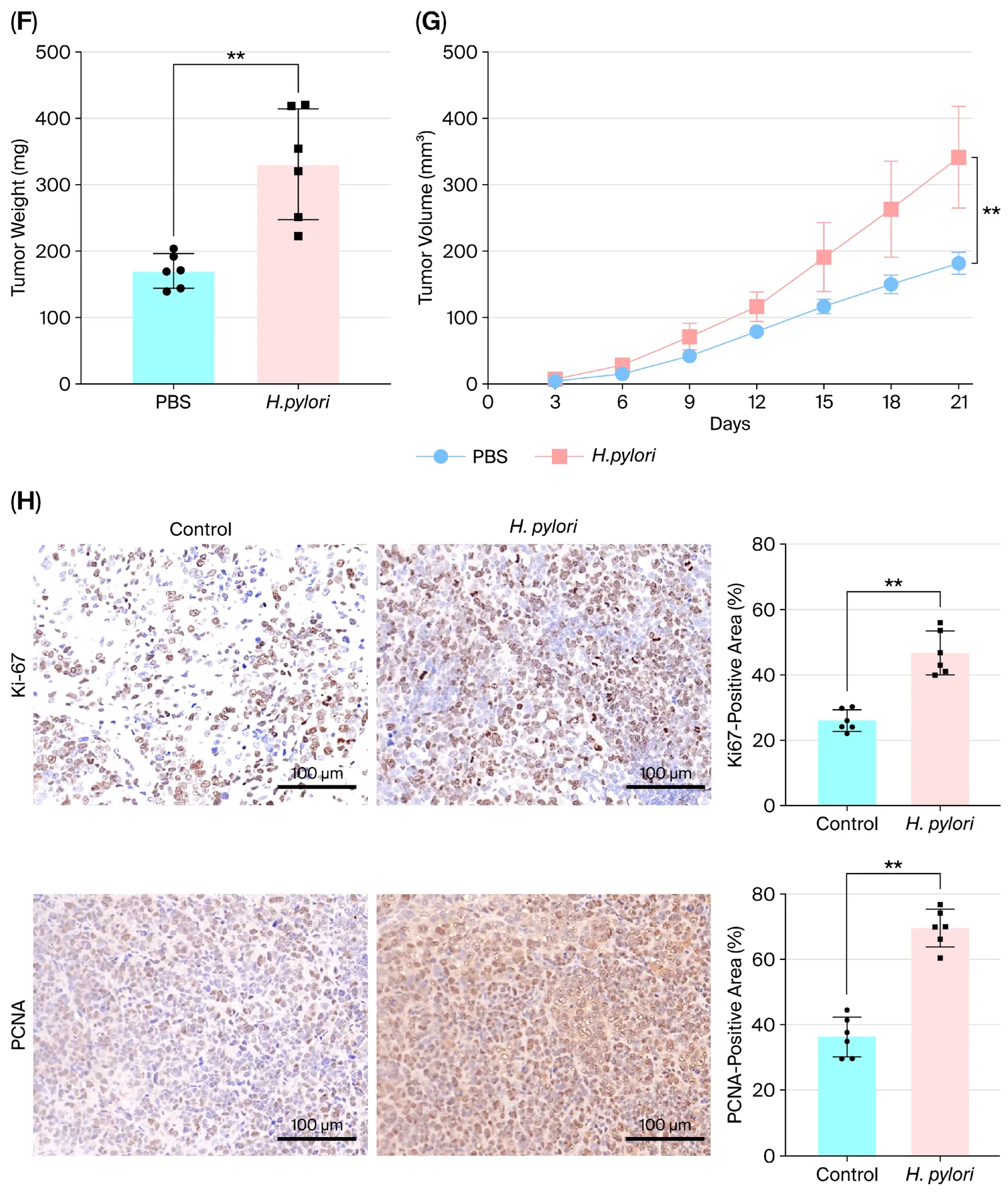
Viable *H. pylori* interacts with HCC cells and enhances subsequent tumor growth in vivo. (**A**) Representative Columbia blood agar plates from adhesion and gentamicin protection assays using Huh7 and Hepa1-6 cells cocultured with *H. pylori* SS1 at a multiplicity of infection (MOI) of 100. The indicated dilutions were used for CFU enumeration. (**B**) Quantification of viable *H. pylori* recovered from Huh7 and Hepa1-6 cells in the adhesion and gentamicin protection assays. (**C**) Representative transmission electron micrographs showing *H. pylori* closely associated with HCC cells. Yellow arrowheads indicate *H. pylori*. Scale bars = 1 μm. (**D**) Experimental scheme for the syngeneic tumor model. Hepa1-6 cells were exposed to *H. pylori* for 24 h, extensively washed, and subcutaneously implanted into C57BL/6J mice. Created in BioRender. Jun-Peng, Z. (2026) https://BioRender.com/ybdfuwe. (**E**) Representative excised tumors. Scale bar = 1 cm. (**F**) Endpoint tumor weights. (**G**) Tumor growth curves. (**H**) Representative Ki-67 and PCNA immunohistochemical staining and quantification of positive areas. Scale bars = 100 μm. Cell-culture experiments were independently repeated three times, and each animal group contained six mice. The error bars denote the standard deviation (SD). Animal outcomes were analyzed using exact two-sided Mann–Whitney U tests. The significance annotation in panel G refers to the comparison of individual-mouse tumor-volume AUC. ** *p* < 0.01.

We first examined whether viable *H. pylori* SS1 could directly interact with HCC cells. In adhesion assays, viable *H. pylori* were recovered from both Huh7 and Hepa1-6 cells after extensive washing. Gentamicin protection assays further detected viable intracellular bacteria in both cell lines, indicating that *H. pylori* can invade HCC cells (Figure 1A,B). After plating the collected supernatants onto Columbia blood agar and incubating the plates for 48 h, colonies were observed in the control without gentamicin, whereas no *H. pylori* colonies were recovered at any tested dilution after treatment with 200 μg/mL gentamicin for 2 h (Figure S1). Transmission electron microscopy revealed *H. pylori* closely associated with the HCC cell surface, further supporting direct bacterial interaction with HCC cells (Figure 1C).

We next examined whether transient exposure to *H. pylori* was sufficient to influence subsequent tumor growth. Hepa1-6 cells were cocultured with *H. pylori* for 24 h, extensively washed, and then subcutaneously implanted into immunocompetent C57BL/6J mice as shown in Figure 1D. Tumors derived from *H. pylori*-exposed cells showed greater growth over the observation period, with a higher individual-mouse tumor-volume AUC than controls (Figure 1E,G). Endpoint tumor weights were also greater in the *H. pylori*-exposed group (Figure 1F). Histological analysis further showed increased Ki-67- and PCNA-positive areas in tumors derived from *H. pylori*-exposed cells (Figure 1H), indicating enhanced proliferative activity in vivo.

### 2.2. Sterile H. pylori-Conditioned Supernatant Promotes Proliferation of HCC Cells

**Figure 2:**
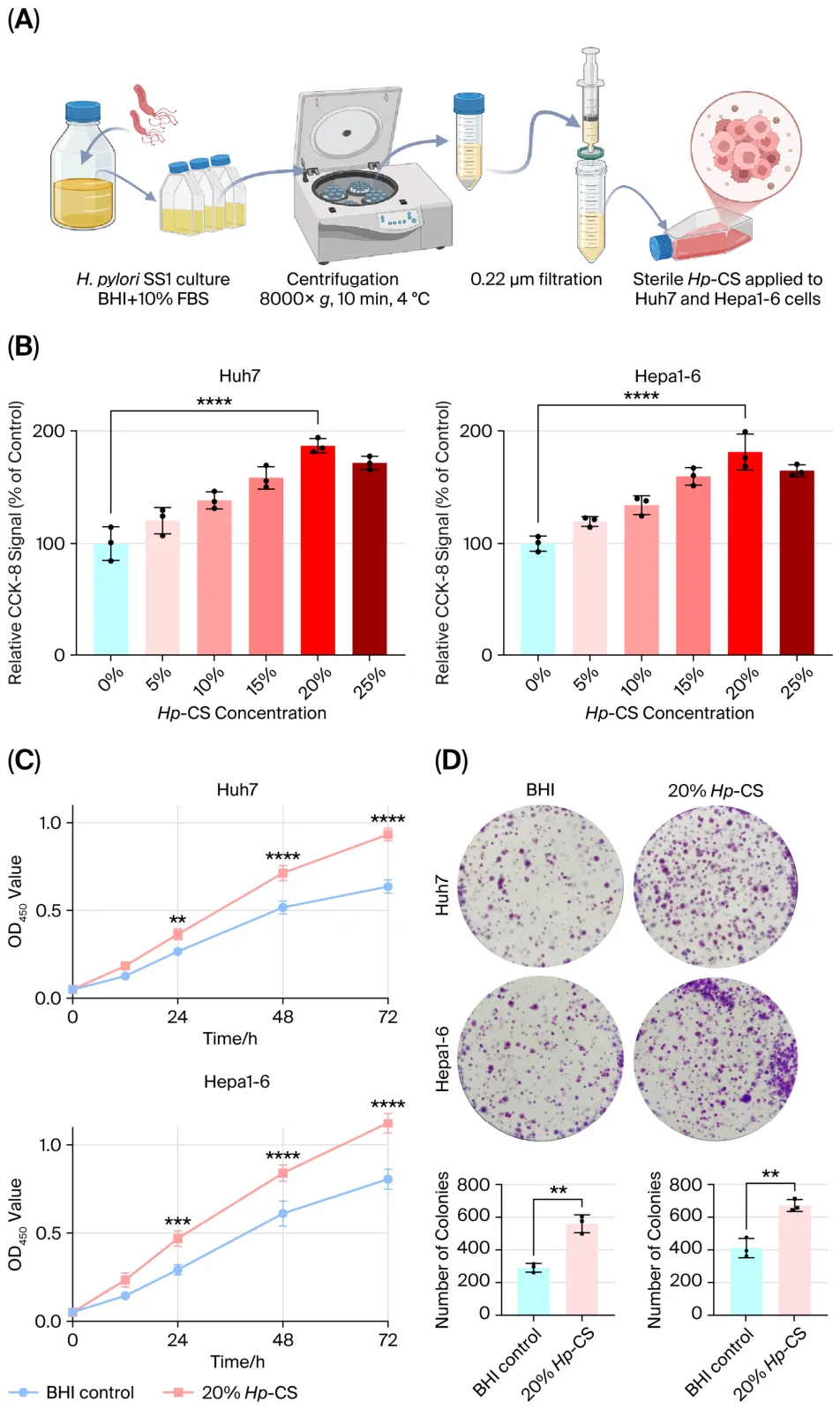
Sterile *H. pylori*-conditioned supernatant promotes proliferation of HCC cells. (**A**) Workflow for preparation of sterile *H. pylori*-conditioned supernatant (*Hp*-CS). *H. pylori* SS1 culture supernatants were centrifuged, passed through 0.22-μm filters, and confirmed sterile by culture. Created in BioRender. Jun-Peng, Z. (2026) https://BioRender.com/mit1fhk. (**B**) Cell metabolic activity of Huh7 and Hepa1-6 cells after treatment with the indicated concentrations of *Hp*-CS. (**C**) CCK-8 metabolic activity of Huh7 and Hepa1-6 cells treated with processed BHI control or 20% *Hp*-CS at the indicated time points. (**D**) Colony formation assay of Huh7 and Hepa1-6 cells treated with processed BHI control or 20% *Hp*-CS. The error bars denote the standard deviation (SD) derived from three independent biological experiments. Dose–response data were analyzed using one-way ANOVA, CCK-8 time-course data using two-way ANOVA, and colony counts using unpaired two-tailed Student’s *t*-test. Multiple-comparison procedures are described in Supplementary Methods S6. ** *p* < 0.01; *** *p* < 0.001; **** *p* < 0.0001.

To identify *H. pylori*-derived factors that may mediate the biological effects observed after transient bacterial exposure, we next examined whether cell-free products released by *H. pylori* were sufficient to alter HCC cell behavior in the absence of viable bacteria. *H. pylori* culture supernatant was centrifuged and filtered to obtain sterile *H. pylori*-conditioned supernatant (*Hp*-CS) (Figure 2A).

Cell Counting Kit-8 (CCK-8) assays showed that *Hp*-CS increased cellular metabolic activity in both HCC cell lines (Figure 2B). Time-course analysis showed progressively increased CCK-8 signals after *Hp*-CS exposure relative to the processed brain heart infusion (BHI) control (Figure 2C). Consistently, colony-formation assays demonstrated increased clonogenic growth in both Huh7 and Hepa1-6 cells after *Hp*-CS exposure (Figure 2D).

In addition, transwell assays showed increased migration and invasion of both Huh7 and Hepa1-6 cells following *Hp*-CS exposure (Figure S2A,B). In Huh7 cells, wound-healing assays showed accelerated wound closure after *Hp*-CS exposure (Figure S2C). Thus, sterile *Hp*-CS was sufficient to promote proliferation-, migration-, and invasion-associated phenotypes in HCC cells in the absence of viable bacteria.

### 2.3. Isolated Hp-OMVs Reproduce Key Proliferation-Associated Effects of Hp-CS In Vitro and In Vivo

**Figure 3:**
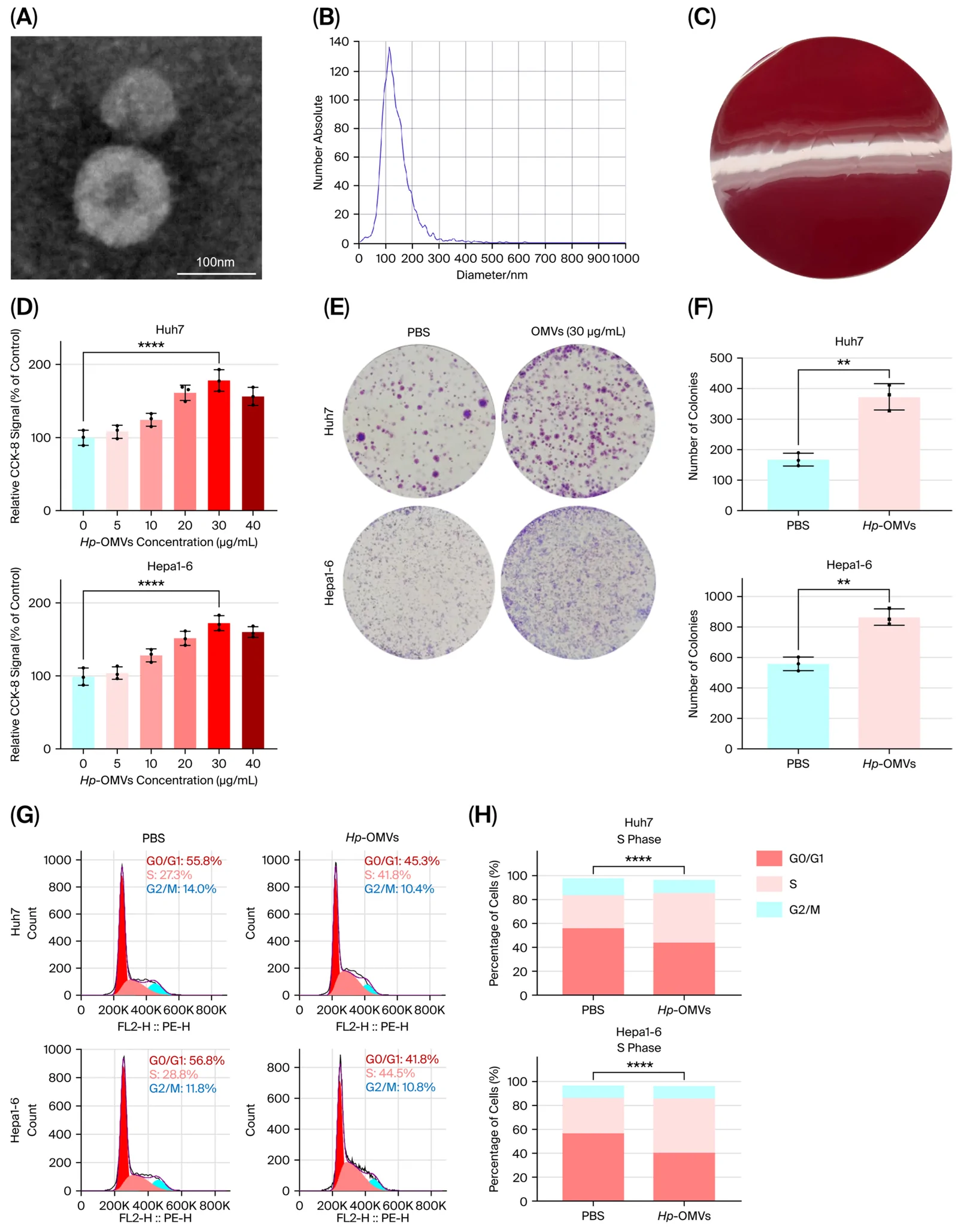

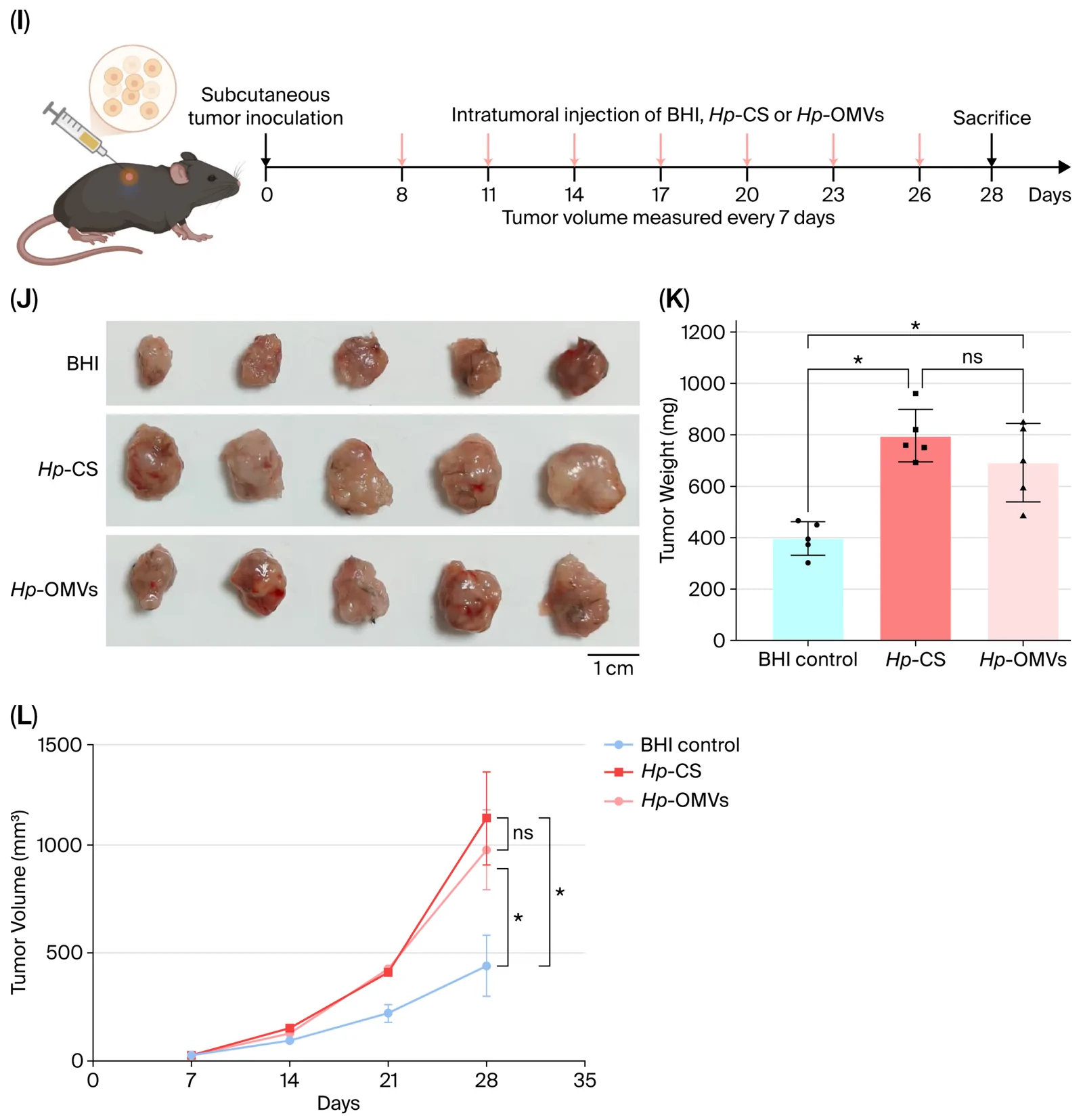
Isolated *Hp*-OMVs reproduce key proliferation-associated effects of *Hp*-CS in HCC cells and tumors. (**A**) Representative transmission electron micrograph of *Hp*-OMVs isolated from *H. pylori* SS1. Scale bar = 100 nm. (**B**) Particle-size distribution of *Hp*-OMVs determined by nanoparticle tracking analysis. (**C**) Representative Columbia blood agar plate showing no recoverable viable bacteria in the final *Hp*-OMV preparation. (**D**) Cell metabolic activity of Huh7 and Hepa1-6 cells after treatment with the indicated concentrations of *Hp*-OMVs. (**E**,**F**) Colony formation assay and quantification of Huh7 and Hepa1-6 cells treated with PBS or 30 μg/mL *Hp*-OMVs. (**G**,**H**) Representative flow-cytometric profiles and cell-cycle distributions after treatment with PBS or Hp-OMVs. Statistical comparisons in (**H**) were restricted to S-phase fractions. (**I**) Experimental scheme for intratumoral administration of processed BHI, 20% *Hp*-CS, or *Hp*-OMVs in mice bearing Hepa1-6 tumors. Created in BioRender. Jun-Peng, Z. (2026) https://BioRender.com/uomnl83. (**J**) Representative excised tumors. Scale bar = 1 cm. (**K**) Endpoint tumor weights. (**L**) Tumor growth curves. In vitro experiments were performed in three independent biological replicates, and each animal group contained five mice. The error bars denote the standard deviation (SD). In vitro data were analyzed using unpaired two-tailed Student’s *t*-test or one-way ANOVA, as appropriate. Animal outcomes were analyzed using Kruskal–Wallis tests followed by Dunn’s multiple-comparisons test. The significance annotations in panel (**L**) refer to comparisons of individual-mouse tumor-volume AUC. * *p* < 0.05; ** *p* < 0.01; **** *p* < 0.0001; ns, not significant.

Having established that sterile *Hp*-CS retained biological activity, we next examined whether *H. pylori*-derived OMVs could reproduce key effects of *Hp*-CS. *Hp*-OMVs were isolated and characterized. Transmission electron microscopy revealed spherical membrane-bound vesicles consistent with bacterial OMVs (Figure 3A), and nanoparticle tracking analysis confirmed a nanoscale particle-size distribution (Figure 3B). No viable bacteria were recovered from the final *Hp*-OMV preparation under the culture conditions used (Figure 3C).

*Hp*-OMV exposure increased the metabolic activity of Huh7 and Hepa1-6 cells (Figure 3D) and enhanced colony formation in both cell lines (Figure 3E,F).

Flow-cytometric analysis showed a G0/G1-to-S phase shift after *Hp*-OMV exposure (Figure 3G,H).

To determine whether these growth-promoting effects were also evident in vivo, mice bearing established Hepa1-6 tumors received intratumoral administration of processed BHI vehicle, *Hp*-CS, or *Hp*-OMVs according to the schedule shown in Figure 3I. Both *Hp*-CS and *Hp*-OMVs enhanced tumor growth compared with vehicle, with no significant difference between them (Figure 3J–L). Thus, isolated *Hp*-OMVs reproduced key proliferation-associated effects of *Hp*-CS, including enhanced HCC cell growth and increased tumor growth in vivo, and additionally promoted G1/S-phase progression.

### 2.4. Viable H. pylori, Hp-CS, and Hp-OMVs Increase CCND1/Cyclin D1 Expression in HCC Cells

**Figure 4:**
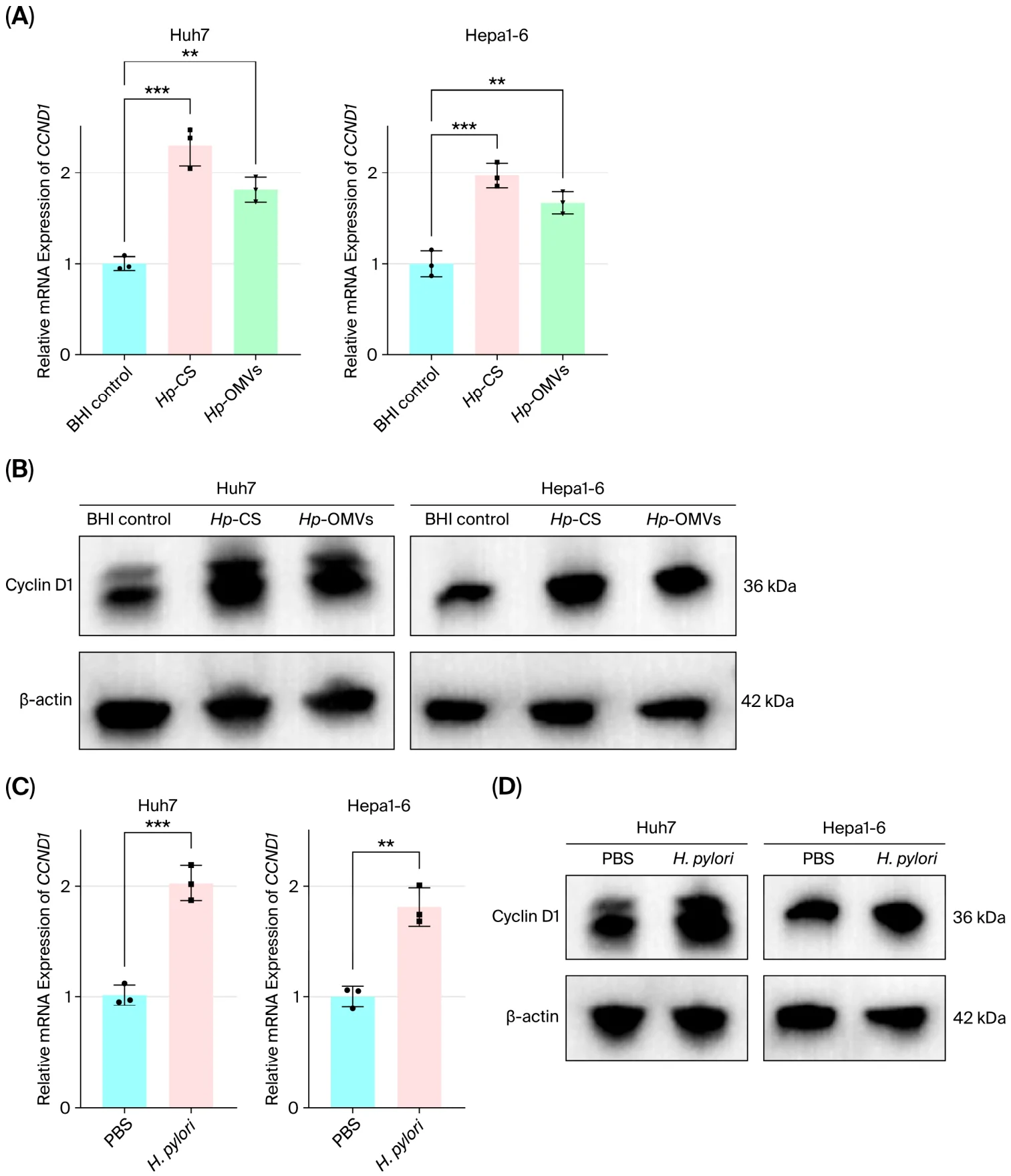
Viable *H. pylori*, *Hp*-CS, and *Hp*-OMVs increase *CCND1*/Cyclin D1 expression in HCC cells. (**A**) Relative *CCND1* mRNA expression in Huh7 and Hepa1-6 cells treated with processed BHI control, 20% *Hp*-CS, or 30 μg/mL *Hp*-OMVs. (**B**) Western blot analysis of Cyclin D1 expression under the same treatment conditions. β-Actin was used as the loading control. (**C**) Relative *CCND1* mRNA expression in Huh7 and Hepa1-6 cells following exposure to viable *H. pylori* SS1 or PBS control. (**D**) Western blot analysis of Cyclin D1 expression following exposure to viable *H. pylori* SS1 or PBS control. β-Actin was used as the loading control. The error bars denote the standard deviation (SD) derived from three independent biological experiments. Statistical significance was assessed using unpaired two-tailed Student’s *t*-test or one-way ANOVA, as appropriate. ** *p* < 0.01; *** *p* < 0.001.

The *Hp*-OMV-induced G0/G1-to-S phase shift prompted us to examine G1/S regulators. We assessed *CCND1*/Cyclin D1 expression after exposure to viable *H. pylori* and its extracellular products. Huh7 and Hepa1-6 cells were treated with processed BHI control, *Hp*-CS, or *Hp*-OMVs. Reverse transcription quantitative PCR (RT-qPCR) showed that both *Hp*-CS and *Hp*-OMVs increased *CCND1* mRNA expression compared with the processed BHI control in both HCC cell lines (Figure 4A). Consistently, Western blot analysis demonstrated increased Cyclin D1 protein expression after exposure to either *Hp*-CS or *Hp*-OMVs (Figure 4B).

We further examined whether the same molecular response occurred after direct exposure to viable bacteria. RT-qPCR showed that viable *H. pylori* SS1 increased *CCND1* mRNA expression in both Huh7 and Hepa1-6 cells compared with PBS controls (Figure 4C). Consistently, Western blot analysis showed increased Cyclin D1 protein expression following viable *H. pylori* exposure (Figure 4D).

Together, these results show that viable *H. pylori*, *Hp*-CS, and isolated *Hp*-OMVs all increase *CCND1* mRNA and Cyclin D1 protein expression in HCC cells.

### 2.5. PI3K Inhibition Attenuates Hp-OMV-Induced Cyclin D1 Upregulation and Proliferative Phenotypes in HCC Cells

Given the established role of PI3K/AKT/mTOR signaling in cell growth and cell-cycle progression, we examined whether this pathway contributed to *Hp*-OMV-induced Cyclin D1 upregulation and associated proliferative phenotypes.

**Figure 5:**
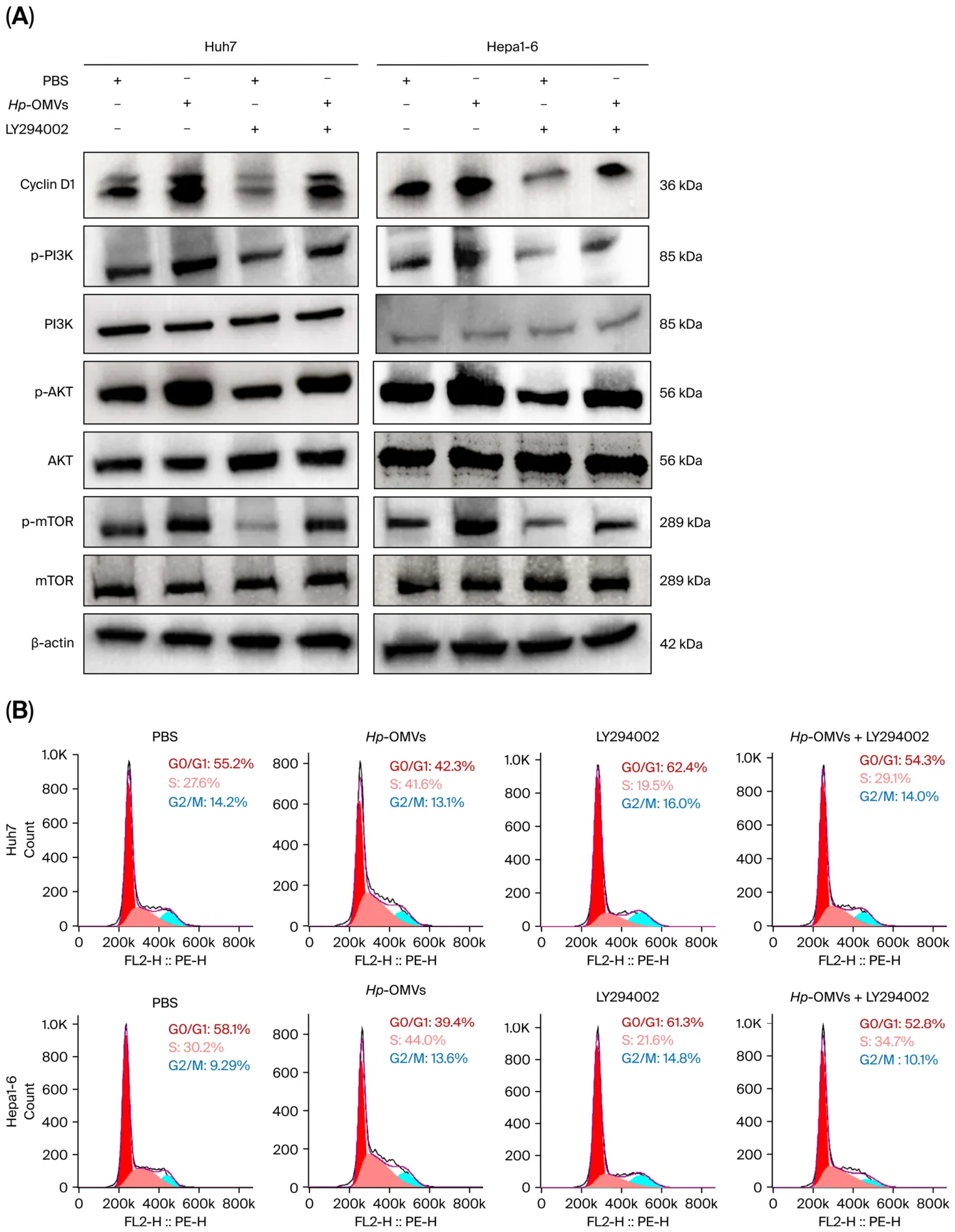

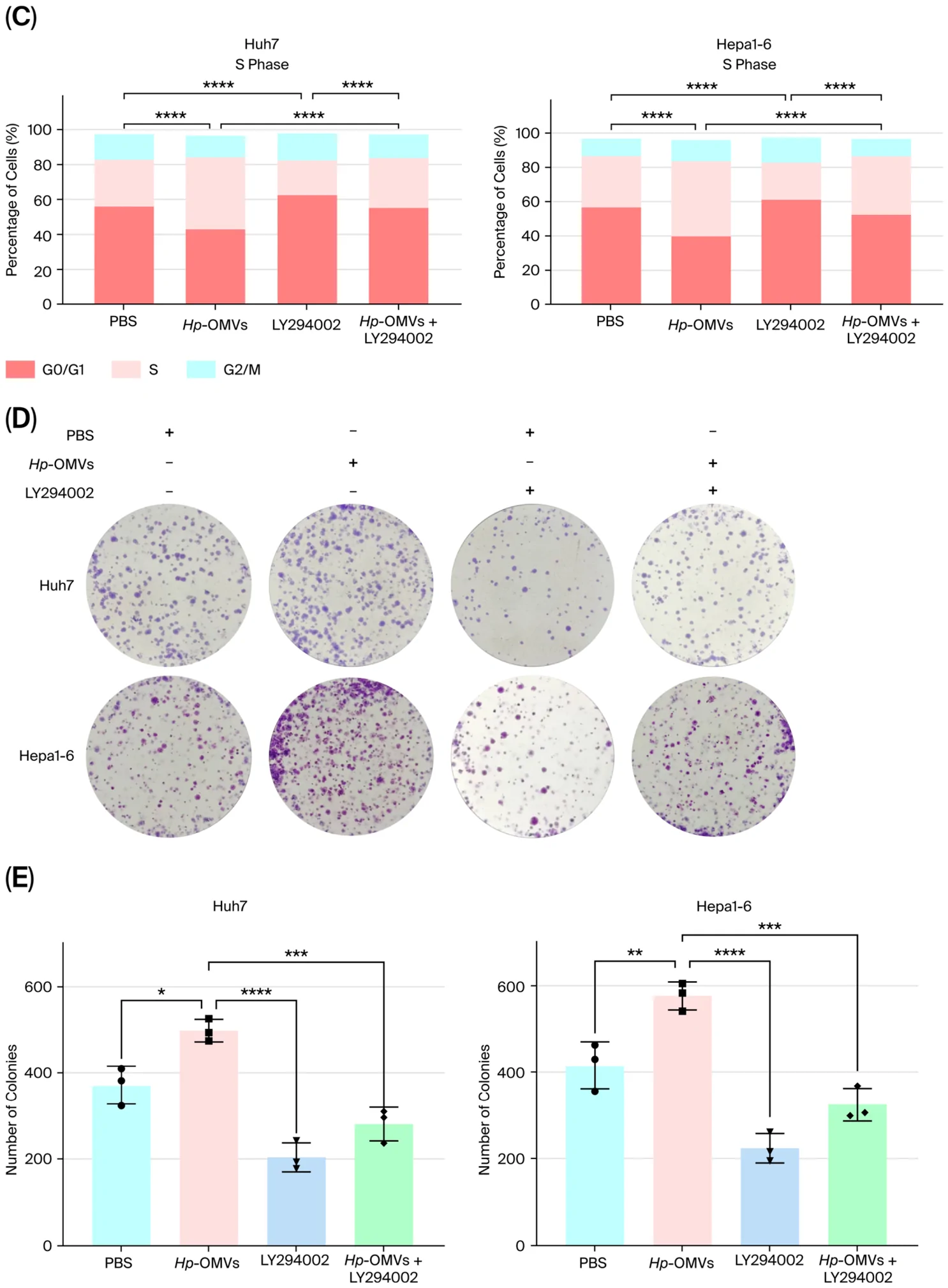
PI3K inhibition attenuates *Hp*-OMV-induced PI3K/AKT/mTOR activation, Cyclin D1 upregulation, cell-cycle progression, and clonogenic growth. (**A**) Western blot analysis of Cyclin D1, phospho-PI3K, total PI3K, phospho-AKT, total AKT, phospho-mTOR, total mTOR, and β-actin in Huh7 and Hepa1-6 cells treated with PBS, 30 μg/mL *Hp*-OMVs, 10 μM LY294002, or *Hp*-OMVs plus LY294002. Cells were pretreated with LY294002 for 1 h before *Hp*-OMV exposure. β-Actin was used as the loading control. (**B**,**C**) Representative flow-cytometric profiles and cell-cycle distributions under the indicated treatment conditions. Statistical comparisons in (**C**) were restricted to S-phase fractions. (**D**,**E**) Colony formation assay and quantification under the indicated treatment conditions. The error bars denote the standard deviation (SD) derived from three independent biological experiments. S-phase fractions in (**C**) and colony counts in (**E**) were analyzed using one-way ANOVA followed by Tukey’s multiple-comparisons test. * *p* < 0.05; ** *p* < 0.01; *** *p* < 0.001; **** *p* < 0.0001.

Huh7 and Hepa1-6 cells were exposed to *Hp*-OMVs with or without the PI3K inhibitor LY294002. *Hp*-OMV exposure increased phosphorylation of PI3K, AKT, and mTOR and increased Cyclin D1 protein expression in both cell lines. Pretreatment with LY294002 reduced *Hp*-OMV-induced phosphorylation of PI3K, AKT, and mTOR and attenuated the accompanying increase in Cyclin D1 expression (Figure 5A).

PI3K inhibition also attenuated *Hp*-OMV-induced changes in cell-cycle distribution. Compared with *Hp*-OMV exposure alone, LY294002 reduced the *Hp*-OMV-induced increase in the S-phase fraction and shifted the cell-cycle distribution toward G0/G1 phase in both Huh7 and Hepa1-6 cells (Figure 5B,C). In addition, LY294002 attenuated the increase in colony formation induced by *Hp*-OMVs in both cell lines (Figure 5D,E).

These data support a role for PI3K/AKT/mTOR signaling in *Hp*-OMV-induced Cyclin D1 upregulation, G1/S-phase progression, and clonogenic growth in HCC cells.

## 3. Discussion

In the present study, we demonstrated that viable *H. pylori* adhered to and invaded Huh7 and Hepa1-6 cells and that transient bacterial exposure enhanced subsequent tumor growth in vivo. Sterile *Hp*-CS retained biological activity after removal of viable bacteria. Exposure to isolated *Hp*-OMVs reproduced key growth-promoting effects observed with *Hp*-CS and viable *H. pylori* and additionally promoted G1/S-phase progression. Mechanistically, *Hp*-OMV exposure increased PI3K, AKT, and mTOR phosphorylation, whereas PI3K inhibition attenuated *Hp*-OMV-induced Cyclin D1 upregulation, S-phase accumulation, and clonogenic growth. Collectively, these findings identify Hp-OMVs as a biologically active extracellular fraction that promotes HCC cell growth and provide pharmacological support for the involvement of PI3K/AKT/mTOR signaling in this response.

Although *H. pylori* has long been recognized as a gastric carcinogen, accumulating evidence suggests that its biological effects extend beyond the stomach [38,39,40,41]. Previous studies have suggested an association between *Helicobacter* detection and HCC [8,9,16], as well as with cirrhosis and portal hypertension [42]. Anatomically, the gastrointestinal tract is connected with the liver through the portal circulation, and the duodenum is connected to the common bile duct and, via the biliary tree, to the liver. Thus, *H. pylori* from the stomach may reach the liver via the common bile duct or the portal circulation [43]. Nevertheless, whether viable *H. pylori* commonly reaches and persists in the human liver remains uncertain. The ability of sterile *Hp*-CS, and particularly isolated OMVs, to alter HCC cell behavior suggests that bacterial extracellular products can exert biological effects without the simultaneous presence of viable bacteria.

*Hp*-CS contains a complex mixture of bacteria-derived molecules, including proteins, lipopolysaccharide, nucleic acids, and secreted virulence-associated factors. Among bacterial extracellular products, OMVs have emerged as important vehicles for bacteria–host communication. OMVs are nanoscale vesicles released by Gram-negative bacteria that can transport outer membrane proteins, lipopolysaccharide, toxins, enzymes, and nucleic acids into host cells [24,25,44]. Previous studies have shown that *H. pylori*-derived OMVs can deliver virulence-associated molecules, including VacA, CagA, urease subunits, and other immunomodulatory cargos, to host cells [23,25,45]. *Hp*-OMVs have also been implicated in gastric epithelial barrier disruption, inflammatory activation, immune regulation, and gastric disease progression [25,46,47]. In our HCC models, *Hp*-OMVs increased cell growth, promoted G1/S-phase progression, and enhanced tumor growth in vivo. Viable *H. pylori*, *Hp*-CS, and *Hp*-OMVs all increased *CCND1* mRNA and Cyclin D1 protein expression in HCC cells.

For *Hp*-OMVs, this response was accompanied by activation of the PI3K/AKT/mTOR pathway. Although PI3K inhibition reduced *Hp*-OMV-induced Cyclin D1 expression and proliferative phenotypes, the effects were not completely abolished, suggesting that additional signaling pathways may participate in this process. *H. pylori* and its extracellular products have been reported to regulate multiple host signaling pathways involved in cell proliferation and inflammation, including MAPK, NF-κB/STAT3, Wnt/β-catenin, and other inflammation-related signaling pathways [48,49,50,51,52]. These pathways may interact with PI3K/AKT/mTOR signaling or independently influence Cyclin D1 expression and cell-cycle regulation. Therefore, *Hp*-OMV-induced responses in HCC cells are likely regulated by multiple mechanisms.

Despite our findings that *H. pylori* OMVs promote HCC cell proliferation with PI3K/AKT/mTOR activation and Cyclin D1 upregulation, there are limitations that need to be addressed in future studies. First, the specific bacterial components responsible for PI3K/AKT/mTOR activation and Cyclin D1 upregulation remain to be identified. Previous studies have shown that *H. pylori* can activate PI3K/AKT signaling in gastric epithelial cells [37,53,54] and that the virulence factor VacA regulates GSK3β through PI3K/AKT-dependent mechanisms [55]. CagA has also been reported to regulate Cyclin D1 expression and cell-cycle progression in gastric epithelial models [50,56,57]. Clinical evidence further links hepatic *cagA* detection to liver disease severity [58]. Whether VacA, CagA, or other *Hp*-OMV cargo components contribute to the pro-proliferative effects observed in HCC cells remains to be determined. Second, the subcutaneous syngeneic tumor model does not fully reproduce the hepatic microenvironment, and intratumoral administration of *Hp*-CS or *Hp*-OMVs was used to assess their biological activity rather than to reproduce the route of exposure occurring during natural gastric infection. Whether *Hp*-OMVs released during infection can enter the portal circulation or hepatobiliary system and reach the liver at biologically relevant concentrations remains to be determined. Third, clinical studies are needed to determine whether *H. pylori*-derived vesicles or their components can be detected in liver tissues and whether their presence is associated with Cyclin D1 expression, PI3K/AKT/mTOR activity, or tumor behavior.

In summary, Hp-OMVs promote proliferation-associated phenotypes in HCC cells, accompanied by PI3K/AKT/mTOR activation and Cyclin D1 upregulation. The present work highlights *Hp*-OMVs as previously underappreciated mediators linking *H. pylori*-derived extracellular signals to HCC progression and provides new insight into microbe-associated regulation of liver cancer biology.

## 4. Materials and Methods

### 4.1. Cell Culture

The mouse hepatocellular carcinoma cell line Hepa1-6 and the human hepatocellular carcinoma cell line Huh7 were obtained from Procell Life Science & Technology (Wuhan, China). Hepa1-6 and Huh7 cells were cultured in Dulbecco’s Modified Eagle Medium (DMEM; Gibco, Grand Island, NY, USA) supplemented with 10% fetal bovine serum (FBS; Gibco) and 1% (*v*/*v*) penicillin–streptomycin (100× stock; Gibco, Thermo Fisher Scientific, Waltham, MA, USA; Cat. No. 15140-122), yielding final concentrations of 100 U/mL penicillin and 100 μg/mL streptomycin. Cells were maintained at 37 °C in a humidified atmosphere containing 5% CO_2_. Cells in the logarithmic growth phase were used for all experiments. For experiments involving *Helicobacter pylori* coculture or exposure to *Hp*-CS or *Hp*-OMVs, both treated cells and their matched controls were maintained in antibiotic-free medium throughout the experimental treatment period.

### 4.2. Bacterial Strain and Culture Conditions

The *H. pylori* Sydney strain 1 (SS1) used in this study was obtained from the *Helicobacter pylori* Research Laboratory, Marshall Centre for Infectious Disease Research and Training, School of Biomedical Sciences, The University of Western Australia. SS1 has been characterized as *cagA*-positive and *vacA*-positive [59]. Detailed culture conditions are presented in Supplementary Methods S1.

### 4.3. Preparation of H. pylori-Conditioned Supernatant

To prepare *H. pylori*-conditioned supernatant (*Hp*-CS), *H. pylori* strain SS1 was cultured in BHI broth supplemented with 10% fetal bovine serum under microaerophilic conditions for 24 h. The bacterial cultures were centrifuged at 8000× *g* for 10 min at 4 °C to remove bacterial cells, and the collected supernatants were subsequently filtered through sterile 0.22 μm polyethersulfone membrane filters to eliminate residual bacteria. Sterility of the filtrates was confirmed by plating aliquots onto Columbia blood agar plates followed by incubation under microaerophilic conditions. BHI broth supplemented with 10% fetal bovine serum was processed using the same centrifugation and filtration procedures and was used as the control.

### 4.4. Isolation and Characterization of H. pylori-Derived Outer Membrane Vesicles

To avoid FBS-derived extracellular vesicles introduced during broth culture, *H. pylori*-derived outer membrane vesicles (*Hp*-OMVs) were prepared using a plate-based method. Briefly, *H. pylori* colonies grown on Columbia blood agar plates were gently harvested and resuspended in sterile phosphate-buffered saline (PBS). The bacterial suspensions were centrifuged at 10,000× *g* for 25 min at 4 °C to remove bacterial cells and large debris. The resulting supernatants were passed through sterile 0.22 μm membrane filters to remove residual bacteria. *Hp*-OMVs were then pelleted by ultracentrifugation at 110,000× *g* for 2 h at 4 °C.

The morphology of the isolated *Hp*-OMVs was examined by transmission electron microscopy (TEM). Total OMV protein concentration was determined using a bicinchoninic acid (BCA) protein assay kit (Beyotime Biotechnology, Shanghai, China) according to the manufacturer’s instructions. Nanoparticle tracking analysis (NTA) was performed using a ZetaVIEW instrument (Particle Metrix, Inning am Ammersee, Germany) equipped with ZetaView software version 8.05.11.

### 4.5. Cell Metabolic Activity and Colony Formation Assays

Cellular metabolic activity was assessed using a Cell Counting Kit-8 (CCK-8; BIMAKE, Houston, TX, USA). Cells were seeded into 96-well plates at a density of 2 × 10^3^ cells per well, with three replicate wells for each group. At 0, 12, 24, 48, and 72 h after exposure to the indicated experimental conditions, 10 μL of CCK-8 reagent was added to each well and incubated at 37 °C for 1 h. Absorbance was measured at 450 nm using a Synergy H1 microplate reader (BioTek Instruments, Winooski, VT, USA).

For colony formation assays, treated cells were seeded into six-well plates at a density of 2 × 10^3^ cells per well and cultured for 10–14 days. Colonies were washed twice with PBS, fixed with 4% formaldehyde for 10 min, and stained with 0.1% crystal violet solution (Solarbio, Beijing, China) for 10 min. Colony images were captured, and colony numbers were quantified for further analysis. For inhibitor experiments, the four treatment groups were PBS control, 30 μg/mL *Hp*-OMVs, 10 μM LY294002, and *Hp*-OMVs plus LY294002; LY294002 was added 1 h before *Hp*-OMV exposure.

### 4.6. Bacterial Coculture, Adhesion, and Gentamicin Protection Assays

Freshly cultured *H. pylori* was harvested from Columbia blood agar plates, suspended in antibiotic-free culture medium, and cocultured with Hepa1-6 or Huh7 cells at a multiplicity of infection (MOI) of 100. For bacterial adhesion and gentamicin protection assays, cells were cocultured with *H. pylori* for 6 h and then washed thoroughly with sterile PBS to remove non-adherent bacteria.

For the bacterial adhesion assay, washed cells were lysed using sterile distilled water containing 0.5% saponin, and the recovered bacteria were serially diluted and plated onto Columbia blood agar plates for colony-forming unit (CFU) enumeration.

For the gentamicin protection assay, cells were incubated with gentamicin at 200 μg/mL for 2 h to eliminate extracellular bacteria [60,61]. Following antibiotic exposure, cells were washed extensively with PBS and lysed with sterile distilled water containing 0.5% saponin. Intracellular bacteria released from the lysed cells were serially diluted, plated onto Columbia blood agar plates, and cultured under microaerophilic conditions. Colony-forming units were subsequently counted to determine intracellular bacterial recovery. Details of extracellular bacterial recovery assessment and viable *H. pylori* exposure for *CCND1*/Cyclin D1 expression analysis are provided in Supplementary Methods S4 and S5, respectively.

### 4.7. Transmission Electron Microscopy

To observe the interaction between *H. pylori* and HCC cells, Hepa1-6 and Huh7 cells were cocultured with *H. pylori* at an MOI of 100 for 6 h. Cells were washed with PBS and fixed overnight at 4 °C in 2.5% glutaraldehyde prepared in 0.1 M phosphate buffer. Following fixation, samples were post-fixed with 1% osmium tetroxide, dehydrated through a graded ethanol series, infiltrated with epoxy resin, and polymerized. Ultrathin sections approximately 70 nm thick were prepared using an EM UC7 ultramicrotome (Leica Mikrosysteme GmbH, Vienna, Austria), mounted on copper grids, and stained with uranyl acetate and lead citrate. Sections were examined using a transmission electron microscope (JEOL, Tokyo, Japan), and representative images were acquired digitally.

For morphological characterization of *Hp*-OMVs, isolated *Hp*-OMV suspensions were placed onto carbon-coated copper grids, negatively stained with 2% phosphotungstic acid, air-dried, and examined using the same transmission electron microscope.

### 4.8. Reverse Transcription Quantitative PCR (RT-qPCR)

For *CCND1* expression analysis, Huh7 and Hepa1-6 cells were treated with processed BHI control, 20% *Hp*-CS, or 30 μg/mL *Hp*-OMVs for 24 h before RNA extraction. Total RNA was extracted from cells using TRIzol reagent (Invitrogen, Carlsbad, CA, USA). Equal amounts of RNA were reverse-transcribed into complementary DNA (cDNA) using the PrimeScript™ RT Reagent Kit (Takara, Maebashi, Japan). RT-qPCR was performed using SYBR Green Master Mix (Takara, Maebashi, Japan) on a LightCycler^®^ 96 system (Roche, Rotkreuz, Switzerland).

The relative expression level of *CCND1* was normalized to *ACTB*, and fold changes were calculated using the 2^−ΔΔCt^ method. Primer sequences are listed in Table S1. Each sample was analyzed in technical triplicate.

### 4.9. Western Blot Analysis

For Cyclin D1 expression analysis, Huh7 and Hepa1-6 cells were treated with processed BHI control, 20% *Hp*-CS, or 30 μg/mL *Hp*-OMVs for 24 h before protein extraction. For pathway-inhibition experiments, Huh7 and Hepa1-6 cells were assigned to four treatment groups: PBS control, 30 μg/mL *Hp*-OMVs, 10 μM LY294002, and *Hp*-OMVs plus LY294002. Cells in the LY294002-containing groups were pretreated with 10 μM LY294002 for 1 h before *Hp*-OMV exposure. Cells were collected after 24 h of the indicated treatments for protein extraction.

Cell lysates were prepared using RIPA buffer (Cell Signaling Technology, Danvers, MA, USA) supplemented with protease and phosphatase inhibitor cocktails (Epizyme, Shanghai, China). Protein concentrations were determined using a bicinchoninic acid (BCA) protein assay kit. Equal amounts of protein were separated by SDS–PAGE and transferred onto polyvinylidene difluoride (PVDF) membranes.

Details of the primary antibodies used for Western blotting are provided in Table S2. After incubation with the corresponding horseradish peroxidase-conjugated secondary antibodies, immunoreactive bands were visualized using enhanced chemiluminescence detection reagents (4A Biotech Co., Ltd., Beijing, China).

### 4.10. Cell-Cycle Analysis

Cell-cycle distribution was analyzed by flow cytometry. Huh7 and Hepa1-6 cells were assigned to four treatment groups: PBS control, 30 μg/mL *Hp*-OMVs, 10 μM LY294002, and *Hp*-OMVs plus LY294002. For the LY294002-containing groups, cells were pretreated with 10 μM LY294002 (TargetMol, Shanghai, China) for 1 h before *Hp*-OMV exposure. Cells were collected after 24 h of treatment for cell-cycle analysis.

After exposure, approximately 1 × 10^6^ cells were harvested per sample by trypsinization, washed twice with PBS, fixed in pre-chilled 70% ethanol, and stored at 4 °C overnight. After removal of the ethanol, the cells were washed twice with PBS and resuspended in 250 μL of DNA staining solution (YEASEN Biotechnology, Shanghai, China). Following incubation at room temperature for 30 min, cell-cycle distribution was analyzed using a CytoFLEX flow cytometer (Beckman Coulter, Brea, CA, USA). Data were processed and analyzed using FlowJo software version 10.10.0.

### 4.11. Syngeneic Tumor Models

Four-week-old male C57BL/6J mice were obtained from GemPharmatech Co., Ltd. (Nanjing, China). Mice were acclimatized for 24 h before experiments and maintained at 26 °C under a 12 h light/12 h dark cycle, with up to six mice housed per cage. To evaluate the effect of transient *H. pylori* exposure on subsequent tumor growth, Hepa1-6 cells were cocultured with *H. pylori* for 24 h and washed several times with sterile PBS. A total of 6 × 10^6^ cells in 100 μL were subcutaneously injected into the right flank of each mouse. Control mice received the same number of Hepa1-6 cells cultured under identical conditions without *H. pylori* exposure. Six mice were included in each group. Tumor size was measured every 3 days using digital calipers, and tumor volume was calculated as follows: tumor volume = length × width^2^/2.

For intratumoral administration experiments, untreated Hepa1-6 cells (6 × 10^6^ cells in 100 μL) were subcutaneously inoculated into the right flank of each mouse. After tumor establishment, mice received intratumoral injections of processed BHI control, 20% *Hp*-CS, or *Hp*-OMVs suspended in processed BHI according to the experimental schedule shown in Figure 3I. Five mice were included in each group. Each *Hp*-OMV injection contained 1.5 μg of OMV protein, with a total injection volume of 50 μL. Intratumoral injections were administered every 3 days. Tumor size was measured every 7 days using digital calipers, and tumor volume was calculated as follows: tumor volume = length × width^2^/2.

At the predefined experimental endpoints indicated in the corresponding experimental scheme, mice were anesthetized with isoflurane and euthanized by cervical dislocation. Tumors were subsequently excised, photographed, weighed, and fixed for further analysis.

### 4.12. Immunohistochemistry

Paraffin-embedded tumor sections were used for immunohistochemical staining. Sections were incubated with primary antibodies against Ki-67 and PCNA (Table S2), according to the manufacturer’s instructions.

Images were captured using an Olympus BX63 microscope (Olympus, Tokyo, Japan), and the Ki-67- and PCNA-positive areas were quantified using ImageJ software version 1.46r. Five microscopic fields were quantified per tumor, and the measurements were averaged to obtain one value per mouse for statistical analysis.

### 4.13. Statistical Analysis

Data are presented as mean ± SD. In vitro assays were performed in three independent biological experiments, with technical replicates averaged within each experiment. Formal normality testing was not used as a prerequisite for test selection because three independent biological experiments cannot reliably establish the underlying distribution.

Parametric tests were selected according to the experimental design and the continuous nature of the in vitro readouts. Statistical findings were interpreted alongside the magnitude and uncertainty of the estimated effects. Mean differences with 95% confidence intervals for two-group comparisons and η^2^ or partial η^2^ for ANOVA models are reported with the corresponding *p* values in Supplementary Table S3. In vitro comparisons were analyzed using unpaired two-tailed Student’s *t*-tests or ANOVA with the multiple-comparison procedures detailed in Supplementary Methods S6, whereas in vivo comparisons used exact two-sided Mann–Whitney U tests or Kruskal–Wallis tests followed by Dunn’s multiple-comparisons test. Each mouse was the experimental unit. Analyses were performed using GraphPad Prism 9.5.0, with two-sided *p* < 0.05 considered statistically significant.

## 5. Conclusions

Collectively, our findings show that extracellular products derived from *H. pylori* can promote proliferation-associated phenotypes in HCC cells. *Hp*-CS increased *CCND1*/Cyclin D1 expression and promoted HCC cell growth and tumor growth, whereas *Hp*-OMVs reproduced these growth-related effects and additionally promoted G1/S-phase progression. *Hp*-OMV exposure also activated PI3K/AKT/mTOR signaling, and PI3K inhibition attenuated *Hp*-OMV-induced Cyclin D1 upregulation, S-phase accumulation, and clonogenic growth. These findings indicate that isolated *Hp*-OMVs are biologically active extracellular components of *H. pylori* capable of influencing HCC cell behavior, and pharmacological inhibition supports the involvement of PI3K/AKT/mTOR signaling and Cyclin D1 upregulation in their growth-promoting effects.

## Data Availability

The data presented in this study are available from the corresponding author upon reasonable request.

## Supplementary Materials

The following supporting information can be downloaded at: https://www.mdpi.com/article/10.3390/ijms27219573/s1.
