# Supplementary material for: Helicobacter pylori-Derived Outer Membrane Vesicles Promote Hepatocellular Carcinoma Cell Proliferation with PI3K/AKT/mTOR Activation and Cyclin D1 Upregulation
Source: Int J Mol Sci. 2026 Oct 26;27(21):9573. doi: 10.3390/ijms27219573 (PMC13763086; doi:10.3390/ijms27219573)
Supplement: Supplementary file 1 [file ijms-27-09573-s001.zip › ijms-4601347-supplementary.pdf]

Supplementary Materials

Helicobacter pylori-Derived Outer Membrane Vesicles Promote Hepatocellular Carcinoma Cell Proliferation with PI3K/AKT/mTOR Activation and Cyclin D1 Upregulation. Zhou et al., 2026.

Supplementary Figure

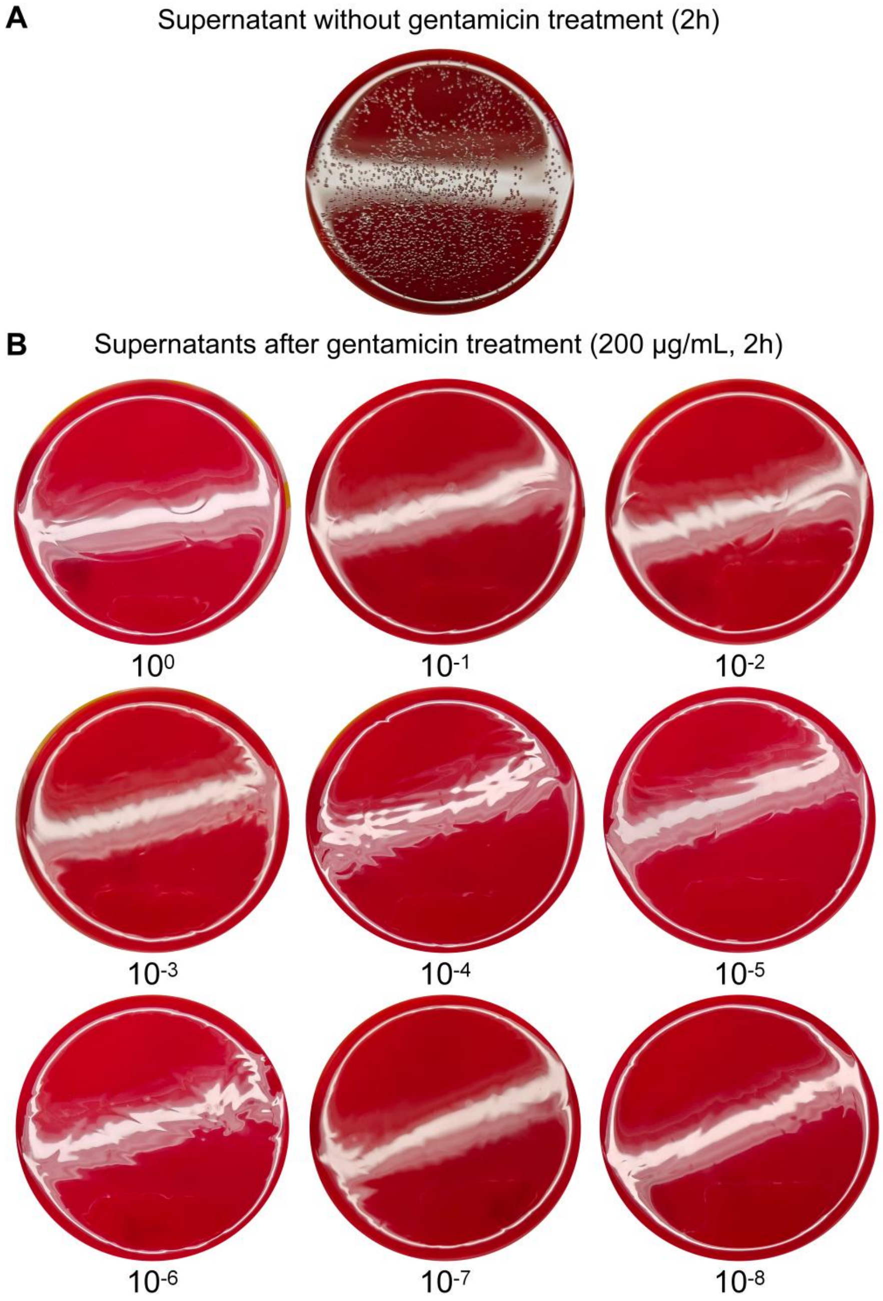

Figure S1. Culture of supernatants following incubation with or without gentamicin.

(A) Culture supernatant collected after 2 h without gentamicin, showing bacterial colony growth. (B) Culture supernatants collected after treatment with 200 μg/mL gentamicin for 2 h were subjected to tenfold serial dilution from 10^0 (undiluted) to 10^{-8} and plated onto Columbia blood agar. Plates were incubated under microaerophilic conditions for 48 h before imaging. No *H. pylori* colonies were observed in the post-treatment supernatants at any dilution.

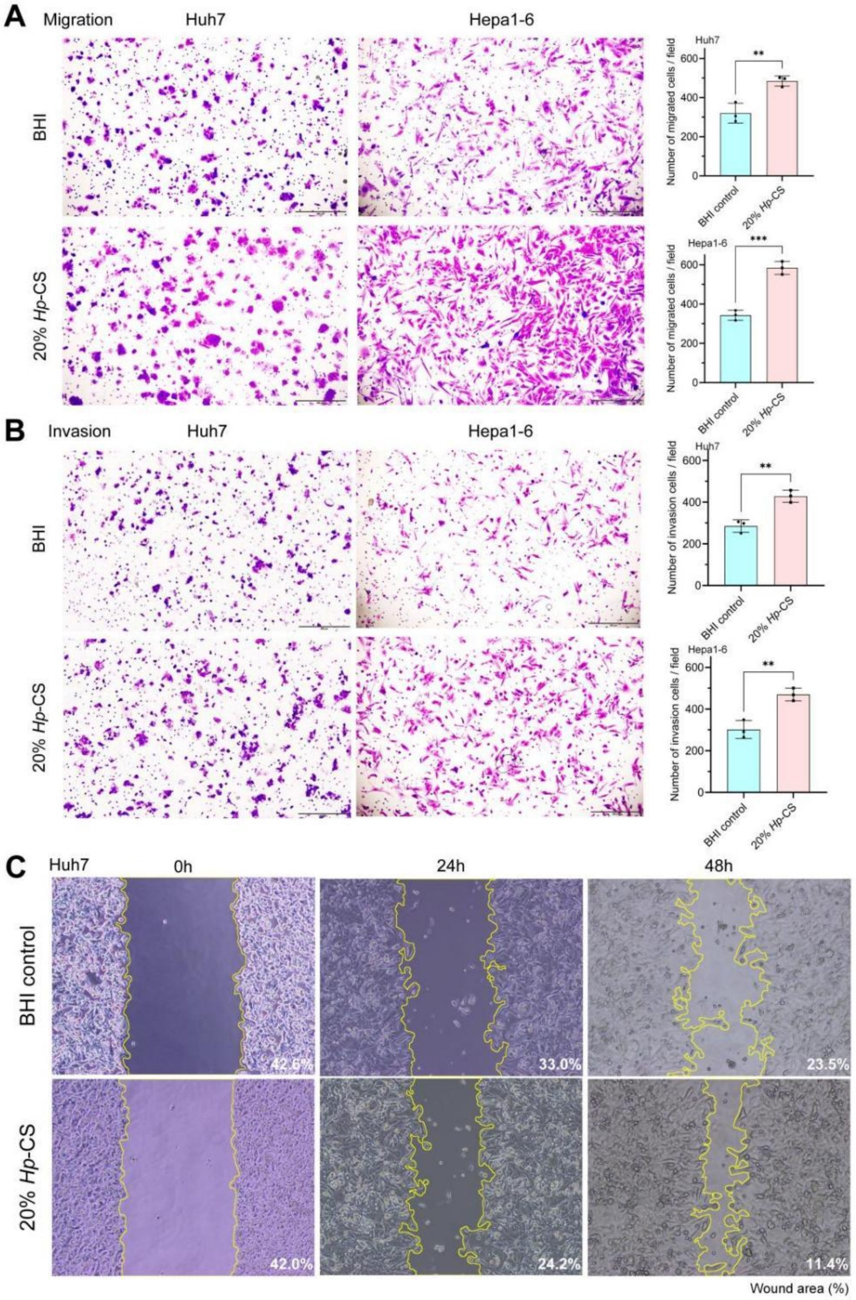

Supplementary Figure S2. Sterile *H. pylori*-conditioned supernatant enhances migration-, invasion-, and wound-closure-associated phenotypes in HCC cells.

(A) Transwell migration assay and quantification of Huh7 and Hepa1-6 cells treated with processed BHI control or 20% *Hp*-CS. (B) Matrigel invasion assay and quantification of Huh7 and Hepa1-6 cells treated with processed BHI control or 20% *Hp*-CS. (C) Wound-healing assay of Huh7 cells treated with processed BHI control or 20% *Hp*-CS at 0, 24, and 48 h. Residual wound area was expressed as the percentage of the total microscopic image area occupied by the cell-free wound region at each time point. Representative images are shown for all three assays. For the Transwell migration and Matrigel invasion assays (A,B), quantitative data are presented as mean ± SD from three independent biological experiments, and comparisons were analyzed using unpaired two-tailed Student's t-tests. **$P < 0.01$; ***$P < 0.001$; ****$P < 0.0001$. For the wound-healing assay (C), residual wound area was quantified and presented descriptively without inferential statistical testing. All three assays were independently repeated three times.

Supplementary Methods

Supplementary Methods S1. Bacterial Culture Conditions

The strain was maintained on Columbia blood agar plates supplemented with 5% defibrinated sheep blood under microaerophilic conditions (approximately 5% O_2, 10% CO_2, and 85% N_2) at 37 °C. Freshly grown colonies were collected and inoculated into brain heart infusion (BHI) broth supplemented with 10% fetal bovine serum for liquid culture. A preliminary growth curve was generated by measuring the optical density at 600 nm (OD_{600}) at the indicated time points. Based on this growth curve, a 24-h culture period was selected to obtain *H. pylori* in the late exponential growth phase. All bacterial cultures used for subsequent experiments were prepared from freshly cultured bacteria.

Supplementary Methods S2. Wound-Healing Assay

Cell migration was evaluated using a wound-healing assay. Huh7 cells were seeded into six-well plates and cultured in complete DMEM containing 10% FBS until confluent monolayers were formed. A uniform scratch was generated using a sterile 200 μL pipette tip, and detached cells were removed by washing twice with PBS. Cells were subsequently cultured in a mixture of 20% (v/v) *Hp*-CS and 80% antibiotic-free DMEM containing 2% FBS; matched controls received processed BHI in place of *Hp*-CS. Images of the wounded areas were captured immediately after scratching (0 h) and at 24 and 48 h using an inverted microscope. The cell-free wound area was measured using ImageJ software and expressed as a percentage of the total image area at each time point.

Supplementary Methods S3. Transwell Migration and Invasion Assays

Cell migration and invasion were evaluated using 24-well Transwell chambers with 8.0 μm pore-size polycarbonate membranes (Corning, NY, USA). Before the Transwell assays, cells were serum-starved for 12 h and then harvested for seeding into the upper chambers.

For migration assays, Huh7 cells (1×10^5 cells) and Hepa1-6 cells (5×10^4 cells) were resuspended in 200 μL of a mixture containing 20% (v/v) *Hp*-CS and 80% antibiotic-free serum-free DMEM and seeded into the upper chambers. Matched controls received processed BHI in place of *Hp*-CS. The lower chambers contained 600 μL of antibiotic-free DMEM supplemented with 10% FBS as a chemoattractant. Huh7 cells were incubated for 24 h, whereas Hepa1-6 cells were incubated for 18 h.

For invasion assays, the inserts were precoated with 50 μL of ice-cold Corning Matrigel Basement Membrane Matrix diluted 1:8 in serum-free DMEM (LDEV-free; Cat. No. 356234, Corning, NY, USA) and incubated at 37 °C for 2 h to allow gel solidification. Huh7 cells (2×10^5 cells) and Hepa1-6 cells (1×10^5 cells) were resuspended in 200 μL of a mixture containing 20% (v/v) *Hp*-CS and 80% antibiotic-free serum-free DMEM and added to the upper chambers. Matched controls received processed BHI in place of *Hp*-CS. The lower chambers contained 600 μL of antibiotic-free DMEM supplemented with 10% FBS. Huh7 cells were incubated for 48 h, whereas Hepa1-6 cells were incubated for 36 h.

After incubation, cells remaining on the upper surface of the membranes were gently removed using cotton swabs. Cells that had migrated or invaded to the lower surface were fixed, stained with 0.1% crystal violet, photographed under a light microscope, and quantified by counting cells in five randomly selected fields per chamber.

The wound-healing, Transwell migration, and Matrigel invasion assays were each performed in three independent biological experiments.

Supplementary Methods S4. Assessment of Extracellular Bacterial Recovery

To assess the effectiveness of gentamicin treatment in eliminating extracellular *H. pylori*, post-treatment culture supernatants were subjected to tenfold serial dilution from 10^0 (undiluted) to 10^{-8} and plated onto Columbia blood agar. Culture supernatant collected after 2 h without gentamicin

served as the positive culture control. Plates were incubated under microaerophilic conditions for 48 h before examination.

Supplementary Methods S5. Viable H. pylori Exposure for CCND1/Cyclin D1 Expression Analysis

For *CCND1*/Cyclin D1 expression analysis, Huh7 and Hepa1-6 cells were cocultured with viable *H. pylori* SS1 at an MOI of 100 for 24 h, with PBS-treated cells serving as controls, followed by RNA or protein extraction for RT-qPCR or Western blot analysis, respectively.

Supplementary Methods S6. Detailed Statistical Analysis

Data are presented as mean ± SD. Unless otherwise indicated, *in vitro* data represent three independent biological experiments. Technical replicates within each independent experiment were averaged and were not treated as independent observations. Formal normality testing was not performed for the *in vitro* datasets because three biological replicates were insufficient for a reliable assessment of the underlying distribution. Accordingly, normality testing was not used as a prerequisite for selecting the *in vitro* statistical tests. Statistical analyses were performed using GraphPad Prism 9.5.0 (GraphPad Software, San Diego, CA, USA). For *in vitro* experiments, comparisons between two groups were performed using a two-tailed unpaired Student's t-test. *In vitro* dose-response comparisons were analyzed using one-way ANOVA followed by Dunnett's multiple-comparisons test against the corresponding control. Other *in vitro* comparisons involving more than two groups were analyzed using one-way ANOVA followed by Tukey's multiple-comparisons test. *In vitro* CCK-8 time-course data were analyzed using two-way ANOVA followed by Šídák's multiple-comparisons test. For *in vivo* experiments, two-group comparisons were analyzed using exact two-sided Mann–Whitney U tests. Three-group comparisons were analyzed using Kruskal–Wallis tests followed by Dunn's multiple-comparisons test, with adjusted *p* values reported for pairwise comparisons. Tumor-volume AUC was calculated separately for each mouse from the actual measurement time points using the trapezoidal rule and compared using the corresponding nonparametric tests. Each mouse was treated as an independent biological replicate. Wound-healing measurements were presented descriptively without inferential statistical testing. A two-sided $p < 0.05$ was considered statistically significant.

Supplementary Tables

Table S1. Primer sequences used for RT-qPCR.

Species	Gene	Forward primer (5′–3′)	Reverse primer (5′–3′)
Human	*CCND1*	TCTACACCGACAACTCCATCCG	TCTGGCATTTTGGAGAGGAAGTG
Human	*ACTB*	CACCATTGGCAATGAGCGGTTC	AGGTCTTTGCGGATGTCCACGT
Mouse	*Ccnd1*	GCAGAAGGAGATTGTGCCATCC	AGGAAGCGGTCCAGGTAGTTCA
Mouse	*Actb*	CATTGCTGACAGGATGCAGAAGG	TGCTGGAAGGTGGACAGTGAGG

Table S2. Primary antibodies used for Western blotting and immunohistochemistry.

Target	Application	Catalog number	Dilution	Supplier
PI3K p85α	WB	HA722522	1:1,000	HUABIO
Phospho-PI3K p85 (Tyr467)/ p55 (Tyr199)	WB	HA721672	1:1,000	HUABIO
AKT1	WB	ET1609-47	1:2,000	HUABIO
Phospho-AKT (Ser473)	WB	ET1607-73	1:5,000	HUABIO
mTOR	WB	ET1608-5	1:5,000	HUABIO
Phospho-mTOR (Ser2448)	WB	HA600094	1:1,000	HUABIO
Cyclin D1	WB	ET1601-31	1:5,000	HUABIO
β-actin	WB	TA-09	1:5,000	ZSGB-BIO
Ki-67	IHC	HA721115	1:5,000	HUABIO
PCNA	IHC	ET1605-38	1:1,000	HUABIO

WB, Western blotting; IHC, immunohistochemistry. HUABIO, Hangzhou, China; ZSGB-BIO, Beijing, China.

Table S3. Effect estimates for the inferentially analyzed *in vitro* experiments.

A. Two-group comparisons analyzed using unpaired two-tailed Student's t-tests

Figure/panel	Endpoint	Cell line	Comparison	Effect estimate	95% CI	*p* value
Figure 2D	Number of colonies	Huh7	*Hp*-CS vs processed BHI control	269.3	(172.1, 366.6)	0.0015
Figure 2D	Number of colonies	Hepa1-6	*Hp*-CS vs processed BHI control	260.3	(148.9, 371.8)	0.0029
Figure 3F	Number of colonies	Huh7	*Hp*-OMVs vs PBS control	206	(129.4, 282.6)	0.0017
Figure 3F	Number of colonies	Hepa1-6	*Hp*-OMVs vs PBS control	306.3	(192.8, 419.9)	0.0017
Figure 3H	S-phase fraction (%)	Huh7	*Hp*-OMVs vs PBS control	13.67	(12.52, 14.82)	<0.0001
Figure 3H	S-phase fraction (%)	Hepa1-6	*Hp*-OMVs vs PBS control	15.93	(13.14, 18.73)	<0.0001
Figure 4C	Relative *CCND1* mRNA expression	Huh7	*H. pylori* vs PBS control	1.013	(0.7166, 1.310)	0.0007
Figure 4C	Relative *CCND1* mRNA expression	Hepa1-6	*H. pylori* vs PBS control	0.81	(0.4894, 1.131)	0.0022
Figure S2A	Migrated cell count	Huh7	*Hp*-CS vs processed BHI control	164	(72.92, 255.1)	0.0075
Figure S2A	Migrated cell count	Hepa1-6	*Hp*-CS vs processed BHI control	240.7	(173.8, 307.6)	0.0006
Figure S2B	Invaded cell count	Huh7	*Hp*-CS vs processed BHI control	143.3	(76.77, 209.9)	0.0039
Figure S2B	Invaded cell count	Hepa1-6	*Hp*-CS vs processed BHI control	168	(83.46, 252.5)	0.0053

Mean differences are defined as the first condition minus the second condition listed in the Comparison column. For S-phase fractions, mean differences are expressed in percentage points.

B. Multi-group and factorial experiments analyzed using ANOVA

Figure/panel	Endpoint	Cell line	ANOVA model	Model effect(s)	Effect size	Omnibus *p* value(s)
Figure 2B	Relative CCK-8 signal (% of control)	Huh7	One-way ANOVA	*Hp*-CS concentration	η^2= 0.9303	<0.0001
Figure 2B	Relative CCK-8 signal (% of control)	Hepa1-6	One-way ANOVA	*Hp*-CS concentration	η^2= 0.9368	<0.0001
Figure 2C	CCK-8 OD450 time course	Huh7	Two-way ANOVA	*Hp*-CS; time; *Hp*-CS × time	partial η^2 = 0.8876; 0.9930; 0.8374	<0.0001; <0.0001; <0.0001
Figure 2C	CCK-8 OD450 time course	Hepa1-6	Two-way ANOVA	*Hp*-CS; time; *Hp*-CS × time	partial η^2 = 0.8428; 0.9893; 0.7117	<0.0001; <0.0001; <0.0001
Figure 3D	Relative CCK-8 signal (% of control)	Huh7	One-way ANOVA	*Hp*-OMV concentration	η^2= 0.9105	<0.0001
Figure 3D	Relative CCK-8 signal (% of control)	Hepa1-6	One-way ANOVA	*Hp*-OMV concentration	η^2=0.9263	<0.0001
Figure 4A	Relative *CCND1* mRNA expression	Huh7	One-way ANOVA	Experimental group (three groups)	η^2=0.9464	0.0002
Figure 4A	Relative *CCND1* mRNA expression	Hepa1-6	One-way ANOVA	Experimental group (three groups)	η^2=0.9329	0.0003
Figure 5C	S-phase fraction (%)	Huh7	One-way ANOVA	Experimental group (four groups)	η^2 = 0.9986	<0.0001
Figure 5C	S-phase fraction (%)	Hepa1-6	One-way ANOVA	Experimental group (four groups)	η^2 = 0.9960	<0.0001
Figure 5E	Number of colonies	Huh7	One-way ANOVA	Experimental group (four groups)	η^2 = 0.9317	<0.0001
Figure 5E	Number of colonies	Hepa1-6	One-way ANOVA	Experimental group (four groups)	η^2 = 0.9385	<0.0001

For one-way ANOVA, η^2 is reported for the overall group effect. For two-way ANOVA, partial η^2 and omnibus *p* values are reported for each main effect and the interaction, in the order listed in the Model effect(s) column. Multiplicity-adjusted post hoc *p* values are indicated in the corresponding figures.
